# Non-Accidental Health Impacts of Wildfire Smoke

**DOI:** 10.3390/ijerph111111772

**Published:** 2014-11-14

**Authors:** Hassani Youssouf, Catherine Liousse, Laurent Roblou, Eric-Michel Assamoi, Raimo O. Salonen, Cara Maesano, Soutrik Banerjee, Isabella Annesi-Maesano

**Affiliations:** 1 Department of Epidemiology of Respiratory and Allergic Disease (EPAR), UMR-S 1136, Institute Pierre Louis of Epidemiology and Public Health, National Institute for Health and Medical Research (INSERM), 27 Rue Chaligny, 75012 Paris, France; E-Mails: hassani.youssouf@upmc.fr (H.Y.); carahenson@gmail.com (C.M.); soutrik.banerjee@upmc.fr (S.B.); 2 Department of Epidemiology of Respiratory and Allergic Disease (EPAR), UMR-S 1136, Institute Pierre Louis of Epidemiology and Public Health, University Pierre and Marie Curie (UPMC)—Sorbonne University, 27 Rue Chaligny, 75012 Paris, France; 3 Laboratory of Aerology, National Center for Scientific Research (CNRS), University of Toulouse, 14 Avenue Edouard Belin, 31400 Toulouse, France; E-Mails: catherine.liousse@aero.obs-mip.fr (C.L.); laurent.roblou@aero.obs-mip.fr (L.R.); eric-michel.assamoi@aero.obs-mip.fr (E.-M.A.); 4 Environmental Epidemiology Unit, National Institute for Health and Welfare, P.O. Box 95, FI-70701 Kuopio, Finland; E-Mail: raimo.salonen@thl.fi

**Keywords:** wildfires emissions, wildfire exposure, health impact, cardiorespiratory disease, particulate matter

## Abstract

Wildfires take a heavy toll on human health worldwide. Climate change may increase the risk of wildfire frequency. Therefore, in view of adapted preventive actions, there is an urgent need to further understand the health effects and public awareness of wildfires. We conducted a systematic review of non-accidental health impacts of wildfire and incorporated lessons learned from recent experiences. Based on the literature, various studies have established the relationship between one of the major components of wildfire, particulate matter (particles with diameter less than 10 µm (PM_10_) and less than 2.5 µm (PM_2.5_)) and cardiorespiratory symptoms in terms of Emergency Rooms visits and hospital admissions. Associations between wildfire emissions and various subclinical effects have also been established. However, few relationships between wildfire emissions and mortality have been observed. Certain segments of the population may be particularly vulnerable to smoke-related health risks. Among them, people with pre-existing cardiopulmonary conditions, the elderly, smokers and, for professional reasons, firefighters. Potential action mechanisms have been highlighted. Overall, more research is needed to better understand health impact of wildfire exposure.

## 1. Introduction

A wildfire is an uncontrolled fire of combustible vegetation that spreads quickly over woodland or brush. Wildfire smoke composition depends on multiple factors, such as fuel type, moisture content, fire temperature and oxygenation, wind conditions and other weather-related influences [1]. A large percentage of wildfires are caused directly or indirectly by human actions [2]. According to a 2010 European Commission report on forest fires, more than 95% of forest fires in many countries in Europe were of human origin and 53% were caused by smoking and fire making in Estonia [2]. However, wildfire spreads is influenced by environmental factors such as high temperatures, drought and temporary dry spells. In northern countries of the boreal hemisphere, fire danger conditions are generally observed in the summer when rain precipitation usually decreases. In tropical areas, fires occur during the dry season, from December to February in boreal hemisphere and from June to September in austral hemisphere. Due to climate change, wildfires have become more problematic for public health and ecosystems in the past decades [2].

It is estimated that wildfire smoke is composed by more than thousands individual chemical compounds and its composition depends on the fuel type, the temperature of the fire, and the wind conditions [1]. Primarily, wildfire smoke is composed of carbon dioxide and water vapor. Other common components of smoke present in lower concentrations are carbon monoxide (CO), formaldehyde, acrolein, polyaromatic hydrocarbons, benzene and particle Matter (PM), namely small particulates suspended in air, which include particles with diameters less than 2.5 micrometers (PM_2.5_) and 10 micrometers (PM_10_). However, about 80%–90% of mass particulate matter produced by wild land is within the fine particles (PM_2.5_) range with high black carbon, organic carbon [3] and brown carbon contents [4].

Many of wildfire emissions can have acute or long term health implications on the exposed populations, according to the official Organizations for health protection [1,5,6]. Among the major components of wildfire smoke, fine particles (PM_2.5_) affects ambient air quality and has various effects on human health [7]. These effects are expected to further increase during wildfire smoke episodes where PM_2.5 _and PM_10_ concentrations above air quality standards can occur [8]. Standard values according to the United States Environmental Protection Agency (USEPA) are fixed in 24 h exposure, to 35 µg/m^3^ for PM_2.5_ and 150 µg/m^3^ for PM_10_ [9]. Mean annual standard values according to the World Health Organization (WHO) are 12 µg/m^3^ for PM_2.5 _and 35 µg/m^3^ for PM_10 _respectively. In our observational data, we have found values 75 times higher than the WHO standard for PM_2.5 _during a wildfire in Spain. Fine particles have been observed to causes changes in lung functions, leading to increases in respiratory and cardiovascular mortality and morbidity including asthma. Fine particles may reach the alveoli, and if not sufficiently cleared in the lungs and greats concentration may enter the bloodstream or remain in the lung, resulting in chronic lung disease such as emphysema. Other wildfire emissions like volatile organic compounds (VOCs) may cause skin and eye irritation, drowsiness, coughing and wheezing, while other like benzene may be carcinogenic [10,11]. 

The health effects of wildfires are less widely known [12]. So far, despite the fact that wildfires take a heavy toll on human health worldwide, few studies reported health effects of wildfires, in particular health effects of trace gases and aerosols emitted by wildfires remain poorly quantified due to uncertainties on wildfire emissions assessment, and the taking into account of their transport and chemical evolution during the long range transport (ozone production, interactions between ozone and aerosols) [11].

Some epidemiological studies have established the relationship between exposure to PM from wildfire smoke and increased visits to hospital emergency rooms and hospital admissions for cardiorespiratory diseases [13,14,15,16,17,18]. Exposure to wood smoke PM_2.5 _from wild land fires and agricultural burning has been linked with cardiovascular effects, including increased cardiovascular mortality, risk of developing cardiovascular disease and risk of myocardial infarction [19]. However, with the exception of two literature reviews [8,11], limited to papers published until January 2012 [8,11,14], data on wildfire related health effects are sparse and many questions remain unanswered. 

The aim of the present article was to contribute to the knowledge on health impacts of wildfires by providing a systematic review of non-accidental health outcomes related to wildfire exposure according to the literature, including subclinical effects and health effect in firefighters in order to understand mechanisms. In the present review, non-accidental has been defined any outcome where there is no identifiable incident, trauma, stress or other mental conditions resulting directly from the fire.

## 2. Methods

### 2.1. Search Strategy

A literature search of online databases (PubMed, ISI, and Google Scholar) up to April 2014 without specifying the start date was performed using the terms, “wildfire smoke exposure”, “bushfire smoke exposure”, “forest fire smoke exposure”, “wildfire emissions”, “bushfire emissions”, “forest fire emissions”, “health effects of wildfire”, “health effects of bushfire”, “health effects of forest fire”. In addition, references of the retrieved articles were examined to identify further relevant articles. A sifting process identified (from study titles, abstracts and the full paper) those studies suitable for inclusion in the review. 

### 2.2. Eligibility Criteria

**Figure 1:**
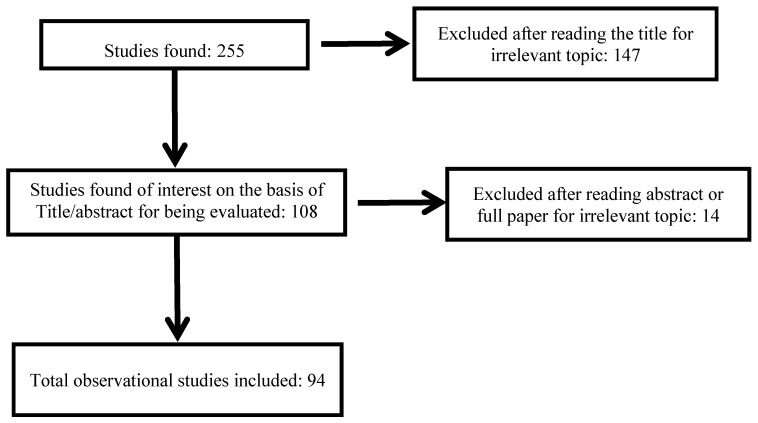
Flow diagram of study selection process.

Published studies were included in this review if they met the following criteria: peer-reviewed original article, review article, population study (cohort, case-crossover, time-series, case–control or cross-sectional study, meta-analysis (none)), written in English and reporting on non-accidental health impacts of wildfire. Figure 1 shows a flow diagram describing the study selection process. 

### 2.3. Data Abstraction

All but the studies meeting the above mentioned criteria were discarded. Data on health impact of wildfires were then systematically summarized in tables where the following characteristics were recorded for each study included: location, publication reference (first author’s last name, year of publication), study population, health outcomes (hospital admissions, mortality), statistical methods, exposure assessment and findings. The percentage increase of health outcomes after exposure to particulate matter was extracted if available. 

## 3. Results

### 3.1. Study Selection

The initial search by key words yielded 255 papers, 94 of which were considered as relevant to the original research question (Figure 1).

### 3.2. Wildfire Exposure Assessment

Several methods have been used to quantify exposure to wildfire emissions in epidemiologic studies investigating health impact of wildfires [11,14]. Self-administrated questionnaires which estimated the exposure to wildfire based on the declaration of the individuals threatened by flames miscalculated real exposure [16] due to report bias in the absence of a quantitative measure [16,20]. Usual objective methods included the number of known wildfires as a proxy for smoke episodes [11,21], the daily burned area as a proxy for daily smoke exposure [14] or more rarely PM measurements to identify single smoke episodes caused by known fires [22]. Air quality monitoring stations estimated the PM concentration from all sources including those emitted by a fire. However in some cases, monitoring stations were not able to monitor an entire wildfire area [13]. Furthermore, the stations are sometimes far from wildfire sources and may be not representative of the burnt area. Recently, wildfire smoke exposure assessment has been estimated through multiple sources of information [14] as ground-based air quality monitoring [23,24], remote sensing through satellite imagery [18,25] and chemical transport models [26,27]. In particular satellite data were used to evaluate wildfire exposure because spatially-resolved particle mass data provided by satellites is superior to using data provided only by the nearest available monitoring station data [13]. In addition, satellites allow detections of plume *etc.* in which case daily variation in urban pollution is not be confounded with smoke exposure. 

### 3.3. Study Design and Statistical Approaches to Evaluate Wildfire Health Effects

There has been considerable variability in study design and statistical approaches used to evaluate the health effects from wildfire smoke exposure. This can challenge the comparison of results, especially in regards to urban background PM and acute toxicity from wildfire. Concerning the study design, different approaches were taken in order to better distinguish the health impacts of air pollution from wildfire smoke to those from other sources of pollution and to better evaluate health outcomes before, during and after the wildfire exposure [13] and in exposed and non-exposed area [13,21]. A lot of statistical approaches were applied, among them paired *t*-tests and multiple regression [28], time-series analysis [29], logistic regression with repeated measures was used to estimate associations with each outcome [13], generalized linear model with negative binomial distribution and Poisson distribution [13], time-series analysis combined with generalized linear Poisson regression models [30]. Poisson distribution was used in several studies to take wildfire smoke exposure and daily health outcomes into account. Other studies use Case-crossover analysis [31] and ecological studies [21,32]. For all statistical methods, potential confounding factors were taken into account in the multivariate models [33]. 

### 3.4. Non-Accidental Health Effects Related to Wildfire

Based on the literature, non-accidental health outcomes that have been found related to wildfire emissions include: (1) all-causes and cardiorespiratory mortality (Table 1), (2) cardiorespiratory morbidity (Table 2), and (3) birth weight. In addition, it is well documented that certain segments of the population are particularly vulnerable to wildfire smoke-related health risks [34]. Among them people with pre-existing cardiopulmonary conditions [35], the elderly [32], smokers and firefighters [20].

#### 3.4.1. Non-Accidental Health Effects at the Population Level

Table 1 and Table 2 present data showing links between wildfire and non-accidental morbidity and mortality.

**Table 1:** Exposure to wildfire and non-accidental mortality.

Location	Authors	Study Period	Population	Health Outcomes	Exposure Assessment/Analytical Methodology	Findings
Sydney	Morgan *et al.* 2010 [24]	1994–2002		All causes mortality, Respiratory mortality and cardiovascular mortality, respiratory diseases admissions	Monitoring stations using TEOM instruments in 8 monitoring sites	PM_10_ was associated with small increase in all causes mortality at lag0 (0.80% CI : −0.24% to 1.86%) but not associated with respiratory mortality or cardiovascular mortality
					Wildfire PM_10_ is calculated by subtracting total PM_10_ by background PM_10_ (due to others sources) calculated as the 30-days moving average of PM_10_ without wildfire	
Denver	Vedal and Duton [36]	2 days fire in Denver 9 June and 18 June 2002	Denver population area (2 millions)	All-cause mortality data for 2001 and 2002	PM was obtained from daily air pollution from network of monitoring sites for Colorado	9 June 2002: PM_10_ and PM_2.5_ peak of 1 h concentration: 372 and 200 mg/m^3^, respectively 18 June 2002: PM_10_ and PM_2.5_ peak of 1 h concentration: 316 and 200 mg/m^3^, respectively No perceptible increases in daily mortality could be attributed to the increase PM concentrations from wildfire
Kuala Lampur	Sastry* et al.* 2002 [37]	April and November 1997	Population of Kuala Lumpur (2.5 millions)	All-cause mortality data from 1994–1997	Daily measurement from Malaysian Meteorological Bureau	PM_10_ > 210 µg/m^3^ is associated with increase of total non-trauma mortality (relative risk = 1.72 for 65–74)
Sydney	Johnston* et al.* 2011 [31]	1997–2004	Population of Sydney	All-cause mortality data from the Australian Bureau of Statistics	Events were defined as days for which the 24 h city-wide concentration of PM_10_ exceeded the 99th percentile	A recent study conducted by Johnston and colleagues in Sydney looked at the effects of bushfires between 1994 and 2007 and mortality. This study revealed that a 5% increase in non-accidental mortality at lag of 1 day (OR 1.05 (95% CI: 1.00–1.10)) was observed on days of high air pollution from bushfire smoke

**Table 2:** Exposure to wildfire and cardiorespiratory diseases.

Location	StudyAuthors	Study Period	Population	Health Outcomes	Exposure Assessment/Analytical Methodology	Findings
North Carolina	Rappold *et al.* 2011 [18]	June 2008	Population of 42 North Carolina counties	Respiratory diseases	Use Aerosol Optical Depth (AOD) measured by satellite GEOS	In the counties exposed significant increase in cumulative RR for asthma (RR = 1.65 (95% CI: 1.25–2.1)), COPD (RR = 1.73 (95% CI: 1.06–2.83)) and pneumonia and acute bronchitis (RR = 1.59 (95% CI: 1.07–2.34))
				Emergency departments visits	AOD scale from 0–2, high density of plume if AOD > 1.25	ED visits of all respiratory diagnosis were elevated in the exposed counties (RR = 1.66 (95% CI: 1.38–1.91))
					Counties with 25% of areas with AOD > 1.25 were defined as exposed to the smoke plume for each day in high-expose window	Significant increase for Emergency department visits for cardiopulmonary symptoms (RR = 1.23 (95% CI: 1.06–1.43)) and heart failure (RR = 1.37 (95% CI: 1.01–1.85))
Southern California	Kunzli *et al.* 2006 [16]	October 2003	873 high school students and 551 elementary-school children from 16 communities in California	Respiratory diseases	Webmail questionnaire to assess smoke exposure and occurrence of symptoms	Prevalence rates of reported outcomes were much higher among individuals with asthma
				Medication usage	Exposure duration were quantified by the number of days of exposure during the two weeks (not at all, 1–2 d, 3–5 d, 6–10 d, all days)	Dry cough, medication and physician visits were more frequently reported by parents of elementary school children. High school students report eye symptoms
				Physician visits		Six or more days of fire smell was significantly associated with all outcomes
						Six days or more of fire smell is associated with more than four-fold higher rates of eyes symptoms, 3 fold dry cough and sneezing 2 for cold, sore throat, wet cough, medication use, physician visits and missed school due symptoms
Southern California	Mirabelli *et al.* 2009 [17]	October 2003	465 high school students from 12 communities	Respiratory diseases	Webmail questionnaire assess smoke exposure and the occurrence of symptoms	Forty percent (186 of 465) of population reported the odor of wildfire smoke at home
					Log-binomial regression to evaluate associations between smoke exposure and fire-related health symptoms	Increase respiratory and eye symptoms with increasing frequency of wildfire smoke exposure
					Ratio of maximum midexpiratory flow to forced vital capacity as marker of airway size	
Three Provinces of Netherlands (Groningen, Friesland and Drenthe)	Greven *et al.* 2011 [20]	12 months	1330 firefighters	General respiratory symptoms	Questionnaire web-based version of European community Respiratory Health Survey questionnaire, added question to identify the number of incidents, the type, the onset, and the duration of symptoms and possible exposure during the incident	OR of general respiratory symptoms were estimated between 1.2 (95% CI: 1.0–1.4) and 1.4 (95% CI: 1.2–1.7) per 25 firesAn inhalation incident is strongly associated with the presents respiratory symptomsOR between 1.7 (95% CI: 1.1–2.7) and 3.0 (95% CI: 1.9–4.7).
				Atopy and bronchial hyper-responsiveness		
Brazilian Amazon Region	Ignotti *et al*. 2010 [32]	2004–2005	Population of Brazilian amazon region	Rates of respiratory hospitalization among children, elderly and intermediate age group and due to childbirth	Annual hours (AH%) of PM_2.5_ > 80 µg/m^3^ AH = (sum of hours with PM_2.5_ > 80 µg/m^3^/sum hours PM_2.5_ is measured in the year) × 100 Use of a coupled aerosol and gas transport model to estimate atmospheric emission Use of satellite observations of fires to obtain several gas and aerosols particles from biomass burnings	1% of increase of the exposure indicator was associated to an increase of 8% of child hospitalization (children < 5 years), 10% increase in hospitalization of elderly, 5% increase of the intermediate age group
Singapore	Emmanuel *et al.* 2000 [38]	1997		Respiratory diseases	PM_10_, PM_2.5_, and other compounds( nitrogen dioxide, ozone, CO) were measured by 15 stations located through the Island linked via public telephone network to a central control station	Air quality was into the unhealthy range (PSI > 100) on 12 days, the highest PSI was 138.94% of particles observed were PM_2.5_ Haze from the Indonesian forest fire was responsible of 30% increase in outpatient attendances
				Outpatient attendances, accident and emergencies, inpatient care, mortality data		Increase in PM_10_ levels from 50 µg/m^3^ to 150 µg/m^3^ was significantly associated with increase of 12% of upper respiratory tract illness, 19% asthma and 26% rhinitis No significant increase in hospitalization or mortality due to the smoke haze
Victoria, Australia	Tham *et al*. 2009 [39]	2002–2003		Hospital admissions, emergency attendances, air quality and meteorological data	Air pollution from the Aplington air quality monitoring station which had the most complete data and was located away from the coast freeways and industrial settings	Daily levels PM_10_ were strongly associated with respiratory emergency department attendances (*p* < 0.001) No association with hospital admissions (*p* = 0.06) After adjusting for confounding effects of maximum temperature and relative humidity, the strongest associations were observed between PM_10_ and daily respiratory emergency department attendances in Melbourne (RR = 1.018, 95% CI: 1.004–1.033, *p* = 0.01)
Vilnius	Ovadnevaite *et al.* 2006 [40]	August–September 2002	The population of Vilnius	Respiratory diseases, bronchial asthma	Air pollution data from Vilnius monitoring network	Significant increase of average hourly values of PM_10_, NO_2_, CO and SO_2_ during several episodes in 2002
						The number of respiratory diseases and bronchial asthma in September (after longer exposure) was up to 20 times higher (by comparison in July) in some regions about 3 times higher all over the city
Australia	Reisen *et al*. 2011 [41]	2005–2008	130 firefighters	Air toxics within the breathing zone of firefighters	One-way analyses of variance, Student *t*-tests	30% of firefighters had a high exposure risk *i.e.*, exposure to hazardous substance, CO, RP, and formaldehyde exceeds the occupational exposure standard (OES) to 5% to 20% of time, 6% had a very high exposure risk *i.e.*, exposure to hazardous substance exceeds OES for more than 20% of time
					CO values were Log-transformed in all tests to meet the assumption of normal distribution of variables	The majority of firefighters (60%) were exposed in low to moderate levels
Galice, Spain	Caamano-Isorna *et al.* 2011 [21]	2006	156 municipalities	Consumption drugs for anxiolytics-hypnotics and drugs for obstructive airway disease (DOADs) for respiratory health	Additive model for time series analysis	Higher consumption of DOADs among pensioners during the months after the wildfires
						The defined DDDs increased by 17 69 DDDs (95%CI: 0.86–34.51) for male pensioners (10.29% increase) *p* < 0.05 in comparison to male pensioners in municipality unaffected. For the female pensioners of the municipality affected, the increase was 12.9% of DDDs (*p* < 0.05)
						For anxiolytics-hypnotics consumption, there was a significant increase in the DDDs among men (pensioners and no pensioners) in affected municipality (15.88% *p* < 0.05 *vs.* 12.2% *p* < 0.05)
Sydney	Jalaludin *et al.* 2000 [15]	January 1994	Children with a reported history of wheezing in the previous 12 months (32 children recruited)	Peak expiratory flow rates (PEFR)	Generalized estimating equation models	After adjusting for the wildfire period and potential confounders, there was no significant association between mean PM_10_ and PEFR Children without bronchial hyperactivity had a significant negative association between PEFR and PM_10_
Southern California	Delfino *et al.* 2009 [13]	October 2003	*n* = 40,856 (hospital admissions)	Respiratroy admissions, cardiovascular admissions	Generalised estimating equation models for Poisson data	Average increases of 70 µg/m^3^ PM_2.5_ during heavy smoke conditions was associated with 34% increase asthma admissions
						The strongest association between wildfire PM_2.5_ and asthma admissions was observed among elderly aged 65–99 (10.1% increase per 10 µg/m^3^ PM_2.5_, 95%CI: 3%–17.8%) and children aged 0–4 (8.3% increase, 95% CI: 2.2%–14.9%) followed by adults aged 20–64 (4.1% 95% CI: 0.5% to 9%), no association for age 5–18
						No evident association between wildfire related PM_2.5_ on cardiovascular admissions
Sao Paulo State Brazil	Abrex *et al.* 2007 [30]	23 March 2003–27 July 2004	Population admitted for asthma in main hospital of Araraquara	Asthma hospital admissions	Time series analysis	Asthma hospital admission during burning period were 50% higher than those observed during the non-burning period (*p* < 0.001)
					Generalized linear Poisson regression models	After stratification to non-burning and burning periods, it was observed that for the same variation of 10 µg/m^3^ in TSP concentration, asthma hospital admissions increased by 9.7% (95% CI: 2.6–17.2) and 12.7% (95% CI: 2.2–24.3) respectively
Brisbane	Chen *et al.* 2006 [23]	1 July 1997–31 December 2000	Patients admitted in Brisbane	Respiratory hospital admissions	generalized linear model with negative binomial distribution	An increase of PM_10_ from low (<15 µg/m^3^) to high level (>20 µg/m) level, is accompanied by an increase of 19% in respiratory hospital admissions for wildfire days *vs.* 13% for background days
Indonesia	Kunii *et al.* 2002 [42]	29 September–7 October 1997	*n* = 543	Respiratory diseases	8 monitoring sites between Jakarta (Java) and Jambi (Sumatra) were used to air quality measurements, Health effects measured by a face to face structured interview	Concentration of CO and PM_10_: very unhealthy and hazardous levels
						Concentration of PAH were 6–14 times higher in the unaffected area, 91.3% of responders had respiratory symptoms due to the haze
						Elderly had a serious deterioration of overall health
Kuching, Malaisia	Mott *et al*. 2005 [35]	1 January 1995–31 December 1998, fire period 1 Augst–31 October 1997	Population of Kuching region in Malaysia (7 hospitals)	Hospitalizations, all causes, respiratory admissions, cardiovasuclar admissions	Comparison of health outcomes in the wildfire period or post-fire period basing on forecasting estimates established from a historical baseline period of 1 January 1995 through 31 July 1997	Increase respiratory hospitalizations specifically for patients with COPD and asthma patients
						Persons over aged 65 year with previous hospital admissions for any cause any cardiorespiratory disease, any respiratory disease or COPD were significantly more likely to be re-hospitalized during the follow-up period in 1997 than in the follow-up period in the pre-fire years of 1995 or 1996.
Australia	Morgan *et al.* 2010 [24]	1994–2002		Respiratory diseases, respiratory mortality and cardiovascular mortality, respiratory diseases admissions	Monitoring stations using TEOM instruments in 8 monitoring sites	A 10 µg/m^3^ increase in wildfire PM_10_ is associated with: 1.24% (95% CI 0.22% to 2.27%) increase in all respiratory diseases admissions (at lag 0) 3.8% (1.4 to 6.26) increase in COPD admissions at lag 25.02 (1.77 to 8.37) increase in adult asthma at lag (0)
					Wildfire PM_10_ is calculated by subtracting total PM_10_ by background PM_10_ (due to others sources) calculated as the 30-days moving average of PM_10_ without wildfire	
Darwin, Australia	Hanigan *et al.* 2008 [43]	April–November 1995–2005		Respiratory diseases admissions	Daily PM_10_ exposure level is determined using the visibility data to build a predictive model	An increase of 10 µg/m^3^ in same-day estimated PM_10_ was associated with 4.81% (95% CI: −1.04%–11.1%) increase in total respiratory admissions
					Data from 2005 and 1995 were used to assess the performance of the model	A strong association of wildfire PM_10_ and respiratory admission among indigenous people than non-indigenous people (15.02%, 95% CI: 3.73%–27.54% *vs.* 0.67%, 95% CI: 7.55%–9.6%)
					Predictive peaks of PM_10_ during 2000 and 2001 were mapped against wildfire activity records for this period	
Central Florida	Sorenson *et al.* 1999 [44]	June–July 1998	All ages	Emergency room visits, hospital admissions	descriptive statistics	Increased emergency-room visits and hospital admissions for asthma and bronchitis during fire period relative to same period in previous year
Malaysia	Brauer, 1998 [45]		All ages	Outpatient visits	Not specified	Increased visits for asthma, upper respiratory tract symptoms, and rhinitis during vegetation fire episode periods of elevated, PM_10_ in Malaysia
Singapore	Chew *et al.* 1995 [46]		Children less than 12 years old	Emergency room visits	Multiple regression analysis	Increased asthma visits with PM_10_ during episode of exposure to biomass burning emissions in Singapore
Denver	Sutherland *et al.* 2005 [47]	June–July 2002	Adult with COPD	Symptoms	Standard descriptive statistics, repeated measurements ANOVA	Significant increase in symptom index (dyspnea, cough, chest tightness, wheezing, sputum production) on two days of elevated PM2.5 (65 μg/m^3^) relative to control days (14 μg/m^3^). Days of elevated PM attributed to fire smoke by satellite imaging
Kelowna and Kamloops Regions British Columbia	Moore *et al.* 2006 [48]	2003	All ages	Physician visits for respiratory, cardiovascular, and mental illness	Particulate matter obtained from monitoring network of the BC Ministry of Water	A 46% to 78% increase in physician visits for respiratory illness during a 3-week forest fire period in Kelowna, British Columbia
Malaysia	Hisham-Hashim *et al.* 1998 [49]	1997	Children	Lung function	Not specified	Decreased lung function in children during vegetation fire episode compared to preepisode measurements
Malaysia	Tan *et al.* 2000 [50]	1997	Adult military recruits	Blood markers of inflammation	Not specified	Bone marrow stimulated to release immature polymorphonuclear leukocytes into blood during period of exposure to forest fire smoke relative to period following smoke exposure
Isfahan rural areas, Iran	Golshan *et al.* 2002 [51]	1–80 years olds	Adults	Asthma medication, lung function, asthmatic and other respiratory symptoms	physician-administered health questionnaire, physical examinations and spirometry in symptomatic cases	Increased prevalence of respiratory symptoms and various asthma indicators, decreased lung function post-rice stubble burning period relative to period prior to burning in three communities in Iran
Darwin, Australia)	Johnston *et al.* 2002 [52]	April–31 October 2000	All Ages	Emergency room visits	Mean atmospheric concentration PM_10_ per cubic metre per 24-h period	Increased asthma visits associated with PM_10_, especially for concentrations exceeding 40 μg/m^3^
California	Duclos *et al.* 1990 [53]	August 1987	All ages	Emergency room visits	descriptive statistics	Increased respiratory visits in communities exposed to fire smoke

##### 3.4.1.1. All-Cause and Cardiorespiratory Mortality

Few studies have evaluated the effects of wildfire emissions on mortality (Table 1). In a study conducted by Vedal and Duton [36], no effect was observed. However, small sample size probably led to the null result. Haenninen [54] showed that Vedal and Duton were not able to detect mortality effects from the observed data because their study could not in any realistic case produce a positive finding, and the expected negative finding should therefore not by any means be considered as evidence of lack of mortality risk from smoke particles. 

In 11 Southern Finnish provinces exposed to an additional population-weighted average PM_2.5 _level of 15.7 µg/m^3 ^from wildfire smoke between 26 August and 8 September 2002, an increase of 10 µg/m^3^ of urban PM_2.5 _was associated with a 0.5% to 2% increase in mortality [55]. A larger study conducted after a widespread series of fires in Indonesia between April and November of 1997 showed high PM_10_ levels (>210 µg/m^3^) associated with total non-trauma mortality [37]. When the authors extended the study period from 1994 to 1996 by using visibility instead of PM_10_ as the model predictor, severely impaired visibility (about 0.9 km, corresponding with the chosen PM_10_ limit value) was associated not only with increased total mortality, but also with increased cardiovascular and infant mortality. Another study conducted by Morgan and colleagues assessed the effects of bushfire smoke on daily mortality and hospital admissions in Sydney, Australia. This study distinguished the effect of bushfire PM_10_ from urban PM_10_. Bushfire PM_10_ was associated with a small increase in all-cause mortality (0.80%) (95% CI 0.24%–61.86%) but not with respiratory or cardiovascular mortality at lag 0. A recent study conducted by Johnston and colleagues in Sydney investigated the effects of bushfires between 1994 and 2007 and mortality. This study revealed that a 5% increase in non-accidental mortality at lag 1 day (OR 1.05, 95% CI: 1.00–1.10) was observed on days with high air pollution from bushfire smoke [31]. Another recent study conducted by Johnston and colleagues estimated the global mortality attributable to smoke from wildfires and daily and annual exposure to PM_2.5_ from fire emissions between 1997 and 2006. The authors combined outputs from a chemical transport model with satellite based-observations of aerosol optical depth (AOD). Study data indicated that the estimated average annual mortality associated with exposure to wild fire smoke was 339,000 deaths worldwide. Regions most affected were sub-Saharan Africa, with 157,000 wildfire related deaths, and South East Asia, with 110,000 wildfire related deaths [56]. This study did have some regional uncertainty in exposure measurements. 

##### 3.4.1.2. Cardiorespiratory Morbidity 

**Figure 2:**
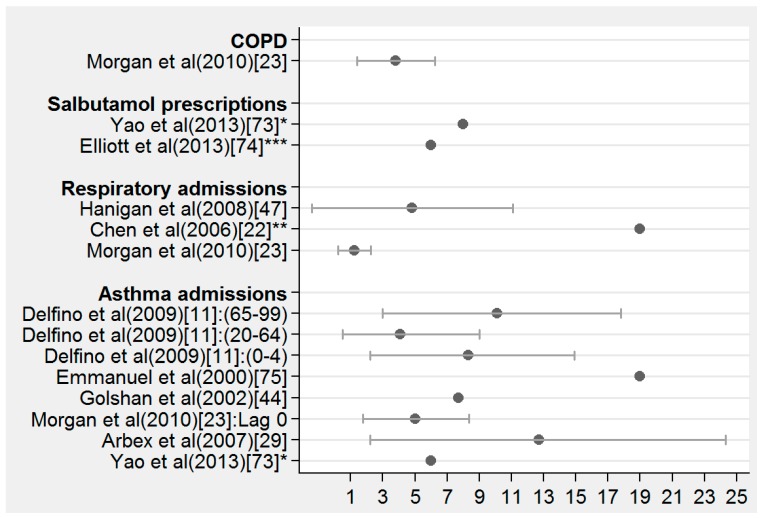
% increase **^1^** of respiratory morbidity outcomes as discussed in the literature per various increases in particle matter (PM) (Mean rate and 95% CI).

Notes: **^1^** Increase in PM is 10 µg/m^3^, if it’s not precised; * increase of 30 µg/m^3^ of PM_2.5_ in Yao* et al.* (2013) [59]; ** increase > 20 µg/m^3^ of PM_10 _in Chen *et al.* (2006) [23]; *** Elliott *et al.* (2013) [60].

Figure 2 presents the percent increase in adverse health outcomes except mortality as discussed in the literature per various increases in PM. According to the study, this ranged from 1 to 19. Increased respiratory symptoms were reported in connection with the 1997 Southeast Asian haze episode [42]. Ninety-one percent of the interviewed persons reported respiratory symptoms. The evidence of increased cardiovascular symptoms, e.g., palpitations (23% of respondents), is noteworthy. In both the USA and the Asian study, wearing masks was associated with decreased occurrence of symptoms. Aditama *et al.* [57] who studied in another analyze the health impact of this same episode of haze in the Southeast Asian by comparing data during September 1997 and June 1998 to data for 2 years earlier between 1995 and 1996 found a significant increase of respiratory outcomes during the forest fire period. This 1997 Southeast Asian haze episode was also investigated by Frankenberg *et al.* [58] who combined data from a population-based longitudinal survey with satellite measures of aerosol levels to assess the impact of smoke on adult health. To account for unobserved differences between haze and nonhaze areas, they compared changes in the health of individual respondents. They found that between 1993 and 1997, individuals who were exposed to haze experienced greater increases in difficulty with activities of daily living than did their counterparts in nonhaze areas. The results for respiratory and general health, although more complicated to interpret, suggest that haze had a negative impact on these dimensions of health.

In the Children’s Health study conducted in California, USA on the effect of smoke from a 2003 wildfire on respiratory symptoms, questionnaires were given to exposed children (age 6–7 years) and teenagers (age 16–19 years). Both respiratory and eye symptoms were observed to increase in children, and those children with the smallest airways had a 2-fold risk of experiencing respiratory symptoms (morning or nighttime dry cough, wet cough, wheezing) compared to other children [17]. Among teenagers, all symptoms (nose, eyes, throat irritation, cough, bronchitis, cold, wheezing, and asthma attack) were increased in relation to smoke episodes [16]. Risk increased monotonically with the number of reported smoky days. There was also increased use of medication and physician’s visits. Since both exposure assessment and evaluation of the health effects were based on questionnaires in the study, it should be noted that participant recall of symptoms might be biased, as they link exposure with worsening health. In the same year, a study of 465 non-asthmatic teenagers affected by 2003 wildfires in Spain revealed that individuals with smaller airways and poorer pre- existing lung function were more vulnerable to smoke effects [10].

Increased emergency department visits for cardiopulmonary symptoms were associated with exposure to smoke from a peat bog wildfire in North Carolina, USA, when smoke covered 42 rural counties. Daily counts of emergency department visits for all respiratory diagnoses were assessed and were found to be elevated in the exposed counties with a relative risk (RR) equal to 1.66 (95% CI: 1.38–1.91). Significant increase in cumulative RR for asthma (RR = 1.65, 95% CI: 1.25–2.1), COPD (RR = 1.73, 95% CI: 1.06–2.83) and pneumonia and acute bronchitis (RR = 1.59, 95% CI 1.07–2.34) was observed in exposed counties. There also was a significant increase in emergency department visits for cardiopulmonary symptoms (RR = 1.23, 95% CI: 1.06–1.43) and heart failure (RR = 1.37, 95% CI: 1.01–1.85) [18]. Morgan *et al.* [24] found that a 10 µg/m^3^ increase in bushfire PM_10_ was associated with the following: a 1.24% increase (95% CI: 0.22%–2.27%) in all respiratory disease admissions and a 5.02% (95% CI: 1.77%–8.37%) increase in adult asthma admissions at lag0; and a 3.8% (95% CI: 1.4%–6.26%) increase in COPD admissions at lag 2.

A considerable impact of wildfire smoke on respiratory and cardiovascular health outcomes was established in British Columbia, Canada, when numerous fires burned in 2003. The association over a 92-day study period were examined between respiratory and cardiovascular physician visits and hospital admissions and three measures of smoke exposure: (1) tapered element oscillating microbalance (TEOM) based air quality PM monitoring, (2) smoke-related PM_10_ from a California puff (CALPUFF) dispersion model, and (3) smoke exposure metric for plumes visible in satellite images [61]. Respiratory physician visits were positively associated with all exposure metrics. A 30 µg/m^3^ increase in total PM_10_ based on TEOM was found associated with increased respiratory physician visits (OR = 1.05, 95% CI: 1.03–1.06), asthma specific visits (OR = 1.16, 95% CI: 1.09–1.23) and respiratory related hospital admissions (OR = 1.15, 95% CI: 1.0–1.29) [61]. Limitations of the study included the TEOM air quality monitors measuring PM_10_ from all sources. While PM_10_ related to smoke was obtained with the help of the CALPUFF model, this method performs poorly under low wind conditions, when fires are typically smoldering and PM_10_ levels are high [61].

Elevated respiratory symptoms have also been associated with the burning of agricultural waste. In Winnipeg, Canada, a group of people with mild to moderate airway obstruction received a symptom questionnaire three weeks after a smoke episode caused by burning of straw and stubble. Forty-two percent of subjects reported having new or exacerbated symptoms (cough, wheezing, chest tightness, shortness of breath) during the smoke episode, and 20% reported having breathing trouble [62]. In Iran [51], burning of residues from rice farming was associated with the increased prevalence of respiratory symptoms, e.g., asthma attacks, use of asthma medication, and cough. Symptomatic subjects underwent spirometric testing. Both FEV1 (forced expiratory volume in 1 sec) and PEFR (peak expiratory flow rate) were decreased in association with the rice burning episode. 

In Brazil, wildfires have been associated with increased rates of respiratory hospital admissions and emergency room visits (Table 2). Asthmatics in particular, but also those with COPD, are especially susceptible. Because levels of particulate air pollution are clearly more elevated during smoke episodes than the levels of gaseous pollutants, it is reasonable to assume that observed health effects are primarily due to PM. Unfortunately, very little information is available on any link between wildfire PM and cardiovascular hospital admissions. In some studies, effects have been observed [53,63], while in others the effects were stronger for respiratory admissions [13,43]. 

Using the incidence of daily counts of hospital admissions for respiratory and cardiovascular diagnoses Crabbe *et al.* [64] calculated the risk of hospitalization from wildfire exposure with respect to PM_10_, in Darwin, Australia. The results suggest that respiratory admissions were associated with exposure to PM_10_ with a lag of 1 day when adjusted for flu and other confounders (RR = 1.025, 95 % CI: 1.000–1.051, *p* < 0.05). This effect is strongest for exposure to fine particulate matter concentrations (RR = 1.091, 95 % CI: 1.023–1.163, *p* < 0.01) when adjusted for flu. Respiratory admissions were also associated with black carbon concentrations recorded the previous day (RR = 1.0004, 95 % CI: 1.000–1.0008, *p* < 0.05), which did not change strength when adjusted for flu. Cardiovascular admissions had the strongest association with exposure to same-day PM and highest RR for exposure to fine particulate matter when adjusted for confounders (RR = 1.044, 95 % CI: 0.989–1.102).

Martin *et al.* [65] examined the association between validated bushfire smoke pollution events and hospital admissions in three eastern Australian cities from 1994 to 2007. Smoke events were defined as days on which bushfire smoke caused the 24-h citywide average concentration of airborne particles to exceed the 99th percentile of the daily distribution for the study period. They used a time-stratified case-crossover design to assess the association between smoke events and hospital admissions and compared health outcomes on event days with non-event days. This study showed that in Sydney, events were associated with a 6% (OR = 1.06, 95% CI: 1.02–1.09) same day increase in respiratory hospital admissions. Same day chronic obstructive pulmonary disease admissions increased 13% (OR = 1.13, 95% CI: 1.05–1.22) and asthma admissions by 12% (OR = 1.12, 95% CI: 1.05–1.19). Events were also associated with increased admissions for respiratory conditions in Newcastle and Wollongong.

Delfino* et al.* [13] analyzed the relationship between respiratory and cardiovascular hospital admissions and exposure to the southern California wildfires of 2003. Analyses were performed in terms of PM exposure and wildfire period. They found that several respiratory symptoms increased in relation with PM_2.5_ during the wildfire period. For example, asthma admissions across all ages increased by 4.8% (95% CI: 2.1%–7.6%) in relation to PM_2.5 _during the wildfire period. The strongest wildfire-related PM_2.5 _associations with asthma admissions were for the elderly, ages 65–99 years (10.1% increase), and children ages 0–4 years (8.3%), followed by adults ages 20–64 years (4.1%). A small relative increase in admission rates for total cardiovascular outcomes in people aged 45–99 years in relation to PM_2.5_ during the fires was observed. Considering the wildfire period, there were significantly increased risks for all respiratory hospital admissions after the fires compared with the pre-fire period. Admissions increased for all ages by 17% (*p* < 0.001), and in age groups 5–19 years by 37% (*p* < 0.008) and 65–99 years by 15% (*p* < 0.004). Respiratory admissions concerned were for asthma, acute bronchitis and bronchiolitis and pneumonia. There was a 6.1% increased risk of combined cardiovascular admissions (*p* < 0.05), and an 11.3% increased risk of congestive heart failure admissions after the fires (*p* < 0.06). However, risk of cardiovascular admissions was lower during the fires by 4.4%. A recent reviews through 2010 [8] and 2012 [12] provides a background for respiratory symptoms from wildfire smoke in different regions in the world. As discussed in this review, although using different methods, all studies looking at Emergency Department presentations in relation to a wildfire smoke event have found associations and most studies have also found an association with hospital admissions. However, only a few studies have distinguished between the effects of wildfire PM_10_ and background PM_10_. These studies suggest that PM_10_ from wildfire smoke is at least as toxic as urban PM_10_, but more research is needed.

Some studies used modeling to develop quantitative assessment and prediction of respiratory health outcomes as hist relates to the location and timing of wildland fire emissions relevant for application to future wildfire scenarios. This approach were used by Thelen *et al.* [66] to study of the impacts on respiratory health of the 2007 wildland fires in and around San Diego County. Using coupled empirical and deterministic models describing PM emissions and atmospheric dispersion linked to spatially explicit syndromic surveillance health data records collected through the San Diego Aberration Detection and Incident Characterization system and a Generalized Additive Modeling (GAM) statistical approach, they found that the model captured the variability in emergency department visits due to several factors by including nine ancillary variables in addition to wildfire PM concentration. The model coefficients and nonlinear function plots indicate that at peak fire PM concentrations the odds of a person seeking emergency care is increased by approximately 50% compared to non-fire conditions (40% for the regional case, 70% for a geographically specific case). The sub-regional analyses show that demographic variables also influence respiratory health outcomes from smoke. Other studies addressed the impact of fire smoke but they considered only indoor settings and some of them experimented health impact of exposure wood smoke under controlled environmental conditions. In these studies significant health effects were reported [67,68,69,70].

##### 3.4.1.3. Wildfire Exposure during Pregnancy and Birth Weight

Few studies analyzed the impact of wildfire exposure during pregnancy. One of them conducted by Holstius* et al.* [71] analyzed the potential impact of maternal exposure to wildfire emissions from the October 2003 California wildfire on birth weight. Potential wildfire exposure windows were defined using MODIS satellite imagery from 21 October to 10 November 2003. Holstius *et al.* used records for singleton term births delivered to mothers residing in California’s South Coast Air Basin (SoCAB) during 2001–2005 (*n* = 886,034) and compared birth weights from pregnancies that took place entirely before or after the wildfire event with those pregnancies that occurred during the wildfire. Census tracts located close to air pollution monitors that measured an average daily PM_10 _level of <40 µg/m^3^ during the fire were classified as low exposure, and census tracts with average daily PM_10 _level >40 µg/m^3^ were classified as high exposure. Of the 886,034 births analyzed, 84.4% (n = 747,590) were unexposed in utero. Of the 138,444 babies that received in utero exposure, 43.5% were exposed in the first trimester, 28.5% were exposed in the second trimester, and 28% were exposed in the third trimester. Adjusted models revealed that mean birth weight was 6.1 g lower (95% CI: −8.7–−3.5) among infants exposed in utero during any trimester compared with unexposed infants. Among those exposed in the third trimester, there was a reduction of 7 g (95%CI −11.8–−2.2). The largest estimated effect was observed in the second trimester, with a reduction of 9.7 g (95% CI: −14.5–−4.8). Kessler performed separate analyzes on the same birth records of babies delivered between 2001 and 2005 in the South Coast Air Basin in the same period and found the same results [72].

##### 3.4.1.4. Subclinical Effects 

In contrast to what has been observed for urban air pollution, there is only limited information on the subclinical effects of particles from wildfires. It has generally been assumed that health effect mechanisms are the same for biomass burning PM as for urban PM (e.g., combustion of fossil fuels). However, during wildfires PM concentrations are higher than those usually observed in urban settings so that a greater effect of PM is expected. Indeed we estimated a PM_2.5 _concentration 75 higher than the WHO annual mean standard in Mangualde in Spain in 2002 (data not shown). The literature reports that biomass particles are at the origin of acute inflammation, oxidative stress and in analogy with biomass burning for domestic cooking, diminished response to infections.

###### 3.4.1.4.1. Animal Models

In a recent study conducted in a mouse lung exposed to PM from wildfire, a very rapid cytotoxicity occurred in pulmonary macrophages and oxidative stress responses are seen after wildfire coarse PM instillation. Indeed, at 1 h after PM instillation, authors observed cytotoxicity because both decreased numbers of viable macrophages and increased dead macrophage percentages as compared to controls were observed. An increase in free isoprostanes, an indicator of oxidative stress, from control values of 28.1 ± 3.2 pg/mL to 83.9 ± 12.2 pg/mL was observed a half-hour after PM instillation [73].

Another study conducted during the 2008 wildfire season in California on mouse bioassay analyzed the Toxicity of PM_10‒2.5_ (PM with mass median aerodynamic diameter>2.5 µm and <10µm) and PM_2.5_ obtained during the time of peak concentrations of smoke in the air and compared with PM samples collected under normal conditions from the region during the month of June 2007. Results showed that concentrations of PM were higher during the wildfire episodes and much more toxic to the lung on an equal weight basis than was PM collected from normal ambient air in the region. Toxicity was manifested as increased neutrophils and protein in lung lavage and by histologic indicators of increased cell influx and edema in the lung [74]. 

###### 3.4.1.4.2. Human Beings and Firefighters

One study having evaluated the effect of air pollution from wildfires on systemic inflammation [50], proposed main pathway from exposure to cardiovascular health effects. Among U.S. servicemen, the 1997 Southeast Asian smoke-haze was associated with an increased neutrophil count in the blood, indicative of a bone marrow response to PM_10_. In a Finnish study, conducted among patients, a 10-day forest fire smoke transport episode from Russia increased median values of IL-12 in ischemic heart disease patients’ plasma by 227% but had no effect on other inflammatory markers such as IL-8, C-reactive protein, fibrinogen, white blood cell count and myeloperoxidase in blood circulation [75]. There are no studies on the possible effects of biomass PM on autonomous nervous function, another health effect mechanism proposed for PM.

###### 3.4.1.4.3. Exposure Trials

Other investigations in a controlled human wood smoke exposure assessed the impact of exposure to wood smoke on systemic inflammation, oxidative stress. Wood smoke exposure was controlled by air filters [67,69,70] and climate controlled chamber [68], under different controlled environmental conditions. Indeed, portable air filters were used in a randomized crossover intervention study of 45 healthy adults exposed to consecutive 7-day periods of filtered and nonfiltered air to investigate the impact of wood smoke exposure and the endothelial function and the underlying role of oxidative stress and inflammation in relation to exposure reductions among healthy adults in a wood smoke impacted community. Air filters reduced indoor fine particle concentrations by 60%. Filtration was associated with a 9.4% (95% CI: 0.9%–18%) increase in reactive hyperemia index and a 32.6% (4.4%–60.9%) decrease in C-reactive protein. Decreases in particulate matter and the wood smoke tracer levoglucosan were associated with reduced band cell counts. There was limited evidence of more pronounced effects on endothelial function and level of systemic inflammation among males, overweight participants, younger participants, and residents of wood-burning homes. However, no associations were noted for oxidative stress markers [67].

In order to test the postulate that healthy volunteers exposed to wood smoke particles would demonstrate evidence of both pulmonary and systemic inflammation, ten healthy volunteers were exposed to filtered air and, 3 weeks or more later, to wood smoke particles. Each exposure included alternating 15 min of exercise and 15 min of rest for a total duration of 2 h. Wood smoke was generated by heating an oak log on an electric element and then delivered to the exposure chamber. Endpoints measured in the volunteers included symptoms, pulmonary function tests, measures of heart rate variability and repolarization, blood indices and analysis of cells and fluid obtained during broncho-alveolar lavage. The results showed that at 20 h after wood smoke exposure, blood tests demonstrated an increased percentage of neutrophils, and bronchial and broncho-alveolar lavage revealed a neutrophilic influx. This study conclude that exposure of healthy volunteers to wood smoke particles may be associated with evidence of both systemic and pulmonary inflammation [69].

Another study assessed the effect of systemic inflammation, oxidative stress and microvascular function after controlled wood smoke exposure, was assessed in a randomized, double-blinded, cross-over study among 20 non-smoking atopic subjects were exposed at rest to 14,220, or 354 μg/m^3^ of particles from a well-burning modern wood stove for 3 h in a climate controlled chamber with 2 week intervals. The investigations have focused on the level of oxidatively damaged DNA, inflammatory markers and adhesion molecules before and 0, 6 and 20 h after exposure. Six hours after exposure, measured microvascular function non-invasively by digital peripheral artery tonometry following arm ischemia was measured. The microvascular function score was unaltered after inhalation of clean air (1.58 ± 0.07; mean ± SEM), low (1.51 ± 0.07) or high (1.61 ± 0.09) concentrations of wood smoke particles in atopic subjects, whereas unexposed non-atopic subjects had higher score (1.91 ± 0.09). The level of oxidatively damaged DNA, mRNA of ITGAL, CCL2, TNF, IL6, IL8, HMOX1, and OGG1 and surface marker molecules ICAM1, ITGAL and L-selectin in peripheral blood mononuclear cells were not affected by inhalation of wood smoke particles. Exposure to wood smoke had no effect on markers of oxidative stress, DNA damage, cell adhesion, cytokines or MVF in atopic subjects [68].

#### 3.4.2. Wildfire Health Effects in Firefighters and Mechanisms

Studies conducted among forest firefighters shed light on the physiological responses to wildfire smoke. Typically, health status right after the work-shift is compared to other time periods, but there is little information on exposure. Reinhardt *et al.* [76] summarized measurements of smoke exposure among wildland firefighters and showed that firefighters can be exposed to significant levels of CO and respiratory irritants, including formaldehyde, acrolein, and respirable particulate matter. Extensive research conducted during forest firefighting in the United States and Australia identified CO and respiratory irritants as the major wildfire pollutants of concern to firefighters [77].

To characterize the acute pulmonary and systemic inflammatory effects of exposure to forest fire smoke, Swiston *et al.* [78], analyzed 52 seasonal forest firefighters recruited before and/or after a day of firefighting. Exposure was assessed by questionnaires and measurement of CO levels. The pulmonary response was assessed by questionnaires, spirometry, and sputum induction. Peripheral blood cell counts and inflammatory cytokines were measured to define the systemic response. The authors found that estimate respirable particulate matter exposure was high (peak levels > 2 mg/m^3^) during firefighting activities. Respiratory symptoms were reported by 65% of firefighters. The percentage sputum granulocytes increased from 6.5% to 10.9% (*p* < 0.02) following firefighting shifts with concurrent increases in circulating white blood cells (5.55 × 10^9^ to 7.06 × 10^9^ cells/L, *p* < 0.0001) and band cells (0.11 × 10^9^ to 0.16 × 10^9^ cells/L, *p* < 0.01). Serum IL-6, IL-8 and MCP-1 levels increased following firefighting (*p* < 0.05). There were no changes in band cells, IL-6, and IL-8 following strenuous physical exertion without firefighting. There was an association between changes in sputum macrophages containing phagocytized particles and circulating band cells (*p* < 0.05). These results showed that acute exposure to air pollution from forest fire smoke elicits inflammation within the lungs as well as a systemic inflammatory response.

In a study conducted to investigate the effect of occupational woodsmoke exposure on inflammatory biomarkers in firefighters, Twelve U.S. Forest Service wildland firefighters at the Savannah River Site, South Carolina, volunteered to give blood samples during four prescribed burns between February and March 2011. Concurrent personal PM_2.5_ and CO monitoring of firefighters was conducted. IL-8 showed a significant cross-work shift difference as indicated by a post/pre-work shift ratio of 1.70 (95% CI: 1.35–2.13; *p* = 0.0012). Concentrations of IL-8, CRP, and ICAM-1 increased in >50% of samples across work shift. Firefighters who lighted fires as opposed to other work tasks had the largest cross-work shift increase in IL-8. A significant cross-work shift increase in IL-8 in blood samples was observed in healthy wildland firefighters working at prescribed burns [79].

In order to analyze the CO poisoning among firefighters, Brotherhood *et al.* [80] assessed the carboxyhemoglobin saturation (COHb%) levels from alveolar CO levels in 24 firefighters working with hand tools and in 12 accompanying scientific observers, before and after firefighting (duration 37–187 min) on 15 experimental bushfires. Carboxyhemoglobin levels increased on average by 0.7% per hour in the firefighters and by 0.3% per hour in the observers that indicate that firefighters are generally unlikely to experience hazardous levels of CO exposure.

Benzene was also measured and found to be well below permissible exposure limits, with the highest concentrations occurring among firefighters working with engines and torches burning petroleum-based fuel. Most of these compounds had a health effects so that smoke inhalation is one of the greatest concerns for firefighter health [81].

Firefighting has been associated with decreased lung function, increased systemic and pulmonary inflammation, and respiratory symptoms [28,78,82]. By measuring pulmonary functions on 24 non-smoking United States Forest Service (USFS) firefighters and 2 current smokers recruited during the dormant winter burn seasons of 2003 and 2004, Adetona *et al.* [83] showed that for a given point in time during the season, each additional day of exposure was estimated to be associated with declines of 24 mL in pre-shift FVC and 24 mL in pre-shift FEV1 (*p* < 0.01). Furthermore, cardiovascular disease is the leading cause of on-duty death among firefighters and a major cause of morbidity [84,85]. According to the 2010 firefighter fatality report of the US Fire Administration, sudden cardiac death accounts for 49% of total firefighter fatalities at work [86]. Generalizability of the results to the general population is limited, however, because firefighters are exposed to fresh combustion particles and gaseous pollutants in the proximity of the fire, whereas populations located further away from fires are exposed to more aged particles and considerably lower levels of pollutants.

A risk assessment of firefighter exposure during prescribed burns and wildfires was conducted in Australia [41]. This study monitored air toxins within the breathing zone of the firefighters and showed that 30% of firefighters were exposed to high levels of a hazardous substance (*i.e.*, CO, respirable particles, formaldehyde) that exceeded the occupational exposure standard (OES) for 5% to 20% of time. Six percent of the firefighters were observed to have very high exposures (exceeding OES for more than 20% of time). However, the majority of firefighters (60%) had low or moderate exposures. Firefighters patrolling on top of ridges of steep burn areas or in downwind sectors tended to have high (44%–62%) or very high (31%–44%) exposures, with a large percentage of very high exposures experienced by supervisors (17%). This study was limited by the fact that monitoring was performed at only a small fraction of the burns conducted each year across Australia and may not be representative of the extensive program of fuel reduction burns carried out each year. Monitoring was restricted to tanker-based crews and to the later stages of campaign fire and did not include the initial more intense stage of the fires or hand crews. Lastly, neither firefighter work experience was taken into account, assuming that less experienced firefighters may endure higher smoke conditions, nor climatic factors influencing the smoke plume. 

In another study, 64 career firefighters from the Fire Emergency Service Authority of Western Australia were allocated one of three filter types (particulate, organic vapor or organic vapor/formaldehyde). Data on spirometer, audiometry, self-reported symptoms and personal air sampling were collected before, during and after exposure to bushfire smoke from prescribed burns. Declines in lung function (Forced Expiratory Volume in One Second (FEV1) and oxygen saturation (SaO_2_)) were demonstrated with exposure. Firefighters in the particulate filter group reported significantly more respiratory symptoms compared to the other filter groups after 60 and 120 min of exposure, respectively (67% and 64%, particle group; 22% and 18%, organic vapor group; 11% and 18%, organic vapor/formaldehyde group) [87].

A screening health risk assessment was performed to assess the upper-bound risks of cancer and noncancer adverse health effects among wildland firefighters performing wildfire suppression and prescribed burn management. Of the 15 substances in smoke that were evaluated, only benzene and formaldehyde posed a cancer risk greater than 1 per million, while only acrolein and respirable particulate matter exposures resulted in hazard indices greater than 1.0. The estimated upper-bound cancer risks ranged from 1.4 to 220 excess cancers per million, and noncancer hazard indices ranged from 9 to 360, depending on the exposure group. These values only indicate the likelihood of adverse health effects, not whether they will or will not occur [88].

Concerning the heart attack among firefighters, it’s was reported by Aisebett *et al*., in a study on wildfire suppression and its impact on firefighters’ health in Australia, that fire agency may not completely illustrate the cardiovascular strains of firefighting. Fire agencies do not collect data on cardiac events that occur after the firefighter has completed their assigned shift. Furthermore, early warning signs such as chest pain/angina, increased fatigue, sensation of indigestion/heartburn, and excessive breathlessness that often precede fatal cardiac events are frequently ignored and not reported. The risk of a cardiac event during physical exertion is increased for individuals who possess two or more cardiovascular risk factors [89]. 

In order to protect firefighters against high particulate exposures which have been demonstrated to decrease lung function among firefighters, Edwards *et al.* [90] demonstrated the feasibility of using small real-time particle sensors to inform wildland firefighters so they may make informed decisions on the use of personal respiratory protection. Using 1 mg/m^3^ as an indicator point for use of appropriately designed respiratory protection; such sensors could help prevent 16% to 74% of particulate exposure during prescribed burns when firefighters assess exposure as low or medium. Adherence to such a guideline for the use of respiratory protection would involve its deployment during 3% to 22% of individual 8-hour shifts. In addition, data-logging sensors would provide a valuable tool for tracking exposure to particulates among wildland firefighters for occupational health monitoring [90].

## 4. Public Awareness 

Even when technical solutions exist to reduce exposure to wildfire smoke, the effectiveness of an intervention largely depends on public awareness and compliance with air pollution advisories. A recent USA study demonstrated that only 10-15% of those aware of public alerts for hot weather and urban air pollution episodes actually changed their behavior [91]. It was the personal perception of poor air quality or high temperature, rather than public advisories that typically led to the change in behavior.

It is unclear how effective public air pollution warning systems are in the case of wildfire smoke. Deterioration of air quality is usually easy to recognize, and a possible link between irritant symptoms with simultaneous high smoke concentrations can be identified. At the same time, there is probably a tendency to consider smoke from wildfires as something “natural” and thus, less harmful than, for example, vehicle exhaust. In a study conducted during a wildfire smoke episode among residents of Hoopa Valley National Indian Reservation in California [35], 66% of those residents who recalled a public service announcement took some action to reduce exposure. Most residents (83%) stayed indoors, while 16% left the area, and only 1% used an air filter, likely because air filters are not common in homes. During the episode, ambient PM_10_ concentrations exceeded the US EPA’s 24-h air quality standard (150 µg/m^3^) for 15 days.

## 5. Discussion 

Wildfires can cause significant health effects both in the population in the immediate vicinity as well as in those far from the fire [12] and several studies have sought to establish the link between exposure to wildfire and health outcomes. Among wildfire emissions, PM_10_ and PM_2.5 _are the most studied as those having various effects on human health [7]. Furthermore, local daily and hourly PM_2.5_ and PM_10_ concentrations can increase dramatically from a wildfire even if it is located hundreds of kilometers away because of long-transportation of the aerosol [38,92]. Different methods have been used to measure wildfire exposure but only the most recent enable us to avoid exposure miss-classification. In terms of health, several studies have found a significant association between PM and respiratory symptoms [16,18,35], increased respiratory hospital admissions [32] and increased emergency department visits. Studies have also found an association between daily mortality from wildfires for all-causes of death, including cardiovascular disease [37]. The effects of exposure to wildfire on cardiovascular disease and mortality, however, have not been sufficiently studied to support general conclusions [19].

Not everyone who is exposed to thick smoke will have health problems. The level and duration of exposure, age, individual susceptibility, including the presence or absence of pre-existing lung or heart disease, and other factors play significant roles in determining whether someone will experience smoke-related health problems [1]. Indeed, the elderly, people with pre-existing cardiopulmonary conditions, smokers and, for professional reasons, firefighters, may experience more severe short-term and chronic symptoms [13,41,47]. In addition, individuals with smaller airways may be more susceptible to the respiratory health effects of wildfire smoke [10]. Some epidemiological studies have observed various health effects among these specific groups [13,19,41,47]. More research is needed to evaluate long term health effects from exposure to wildfires.

Two previous reviews had dealt with health impact of wildfires [10,12]. In contrast with these reviews, our review covered more largely the literature on non-accidental health impact and included firefighters, a vulnerable subgroup exposed to fires for professional reasons. Existing data show that in addition to potential injuries, firefights may be concerned by various non-accidental health effects.

A limitation in all studies is exposure assessment. Even for conclusive studies, there is a large uncertainty on the evaluation of personal exposure because there is difficulty in distinguishing wildfire emissions from emissions due to other sources of pollution [8,11,14]. In addition, although available information suggests that wildfire smoke penetrates readily into indoor environments during air pollution episodes caused by wildfires, this has not been measured objectively. Lastly, there are no published studies on personal exposures to PM from wildfires in community settings. 

Another limitation results from the paucity of statistical methods that have been used to relate wildfire exposure to health conditions. Other methods that could be applied include semi-parametric regressions for estimating associations between day-to-day variations in air pollution and mortality after controlling for confounding [93,94]. Such methods using Generalized Additive Model (GAM) to time-series health outcomes like mortality, morbidity, or hospitalization and studying the impact of PM is well established in the last decade with numerous studies cited in the literature [93,94,95,96]. Hierarchical Models for estimating: National-average relative rates, national-average exposure-response relationship exploring heterogeneity of air pollution effects across the country are also possible [97]. Longitudinal methods have to be implemented to evaluate long term health effects from exposure to wildfires. However these methods which generally imply following individuals over time could be limited by the period and the variation of wildfire exposure area. In summary, various studies have established the relationship between one of the major components of wildfire, PM_10_ and PM_2.5_, and cardiorespiratory in terms of hospital emergency rooms visits and hospital admissions. Associations between wildfire emissions and mortality or other diseases have been less investigated. Prevention against fires has to be implemented to protect the population, in particular the segments of the population vulnerable to smoke-related health risks, such as the elderly, people with pre-existing cardiopulmonary conditions, smokers and firefighters.

## 6. Conclusions

This review has demonstrated the impact of wildfire emissions on public health in terms of non-accidental effects at population level and among vulnerable subgroups. The main challenge for epidemiological studies that evaluate the impact of wildfire emissions during a given event is the estimation of personal exposure. So far, most of the used methods have not distinguished air pollution from wildfires from other source-oriented pollution. Various statistical approaches can be used in the epidemiological studies to take into account this uncertainty. However, so few have been applied. Main health effects of wildfire smoke are cardiorespiratory and associations between exposure to wildfire emissions and increasing of hospital admissions and emergency room visits for respiratory and cardiovascular diseases has been observed in the most studies. Few studies reported on cardiorespiratory mortality. Only a small number of studies found other non-accidental effects like the reduction of birth weight. However, various subclinical effects have been associated to wildfire exposure. Some these effects were tested healthy volunteers using a controlled human exposure, animal exposure or* in vitro* studies. 
